# Global optimization of default phases for parallel transmit coils for ultra-high-field cardiac MRI

**DOI:** 10.1371/journal.pone.0255341

**Published:** 2021-08-06

**Authors:** Maxim Terekhov, Ibrahim A. Elabyad, Laura M. Schreiber

**Affiliations:** Chair of Cellular and Molecular Imaging, Comprehensive Heart Failure Center, University Hospital Wuerzburg, Wuerzburg, Germany; University of Duisburg-Essen, GERMANY

## Abstract

The development of novel multiple-element transmit-receive arrays is an essential factor for improving B_1_^+^ field homogeneity in cardiac MRI at ultra-high magnetic field strength (B_0_ > = 7.0T). One of the key steps in the design and fine-tuning of such arrays during the development process is finding the default driving phases for individual coil elements providing the best possible homogeneity of the combined B_1_^+^-field that is achievable without (or before) subject-specific B_1_^+^-adjustment in the scanner. This task is often solved by time-consuming (brute-force) or by limited efficiency optimization methods. In this work, we propose a robust technique to find phase vectors providing optimization of the B_1_-homogeneity in the default setup of multiple-element transceiver arrays. The key point of the described method is the pre-selection of starting vectors for the iterative solver-based search to maximize the probability of finding a global extremum for a cost function optimizing the homogeneity of a shaped B_1_^+^-field. This strategy allows for (i) drastic reduction of the computation time in comparison to a brute-force method and (ii) finding phase vectors providing a combined B_1_^+^-field with homogeneity characteristics superior to the one provided by the random-multi-start optimization approach. The method was efficiently used for optimizing the default phase settings in the in-house-built 8Tx/16Rx arrays designed for cMRI in pigs at 7T.

## Introduction

The implementation of MRI-scanners, operating at ultra-high field static magnetic fields (UHF of B_0_≥7T, promises a significant increase of both SNR and the spatial resolution (up to ~200μm in-plane) [1] of clinical MR-images. Therefore, despite technical challenges related to both B_0_ and B_1_^+^ fields’ inhomogeneities, the interest in the application of UHF scanners for cardiovascular MRI (cMRI) grows [2–4]. At the Larmor frequency of protons ≈ 300MHz (B_0_≈7T) and electrical permittivity of muscle tissue (ε≈60), the wavelength of $B_{1}^{+}‐\mathrm{field}$ (10-12cm) is smaller than the dimensions of a human thorax. This leads to the establishment of a standing wave regime and creates interferences of a B_1_^+^*-*field across an imaged field-of-view (FOV). Additionally, the homogeneity of the B_1_^+^*-*field suffers from strong differences in the permittivity of intra-thoracic structures, in particular, of the lung parenchyma and cardiac muscle tissue. Thus, the spatial distribution of contrast in MR images becomes essentially heterogeneous rising demand for methods capable to neutralize these negative factors.

During the last decades, multiple elements transmit-receive arrays (mTx-arrays) combined with parallel transmit (pTX) technology became an emerging tool for the improvement of the *B*_*1*_^*+*^*-*field homogeneity in UHF cMRI. Starting with standard birdcage coils with individual control of the phases of the driving ports, the technology development has led to customized arrays with up to 32 transceiver elements [5]. The type of mTX-arrays elements also evolved from classical magnetic loops to electrical dipoles, microstrips, and sophisticated loop-dipole hybrids [5–11]. The novel hardware allows for shaping the optimal spatial distribution of the *B*_*1*_^*+*^*-*field to excite a targeted transversal magnetization with the best possible smoothness of both magnitude and phase within a region-of-interest (ROI). This procedure is usually called “*B*_*1*_^*+*^*-*shimming”. Additionally, using the RF-power amplifier (RFPA) operating in pTX mode allows for driving individual TX-arrays elements dynamically, i.e. with varying magnitude and phase of the RF-pulse waveforms to shape excitation profiles (including 2D and 3D) using different optimization criteria [12–16]. Last but not least, both techniques allow for including safety margins regarding specific adsorption rate (SAR) of electromagnetic energy.

Despite the significant progress in the field of subject-specific static and dynamic B_1_^+^-shimming, it remains to be a quite complex research tool. It requires deep expertise in MR-physics, pulse sequence programming, and optimization methods, to build a pipeline including (i) B_1_^+^ -mapping, (ii) B_1_^+^ -shimming parameters computation taking into account SAR margins, and (iii) integration on the scanner system.

For cardiac MRI additional significant efforts for reliable absolute B_1_^+^-mapping are required to overcome limitations related to breathing and cardiac motion [17]. An important step in the development of mTx-arrays for UHF applications is the optimization and fine-tuning of the coil ensuring reasonable image quality and SAR safety for an average subject without additional adjustment procedures. In particular, this includes finding the default set of phase shifts required for the driving voltages of the individual TX-elements such that a uniform B_1_^+^-distribution can be achieved for the largest possible Field-of-View (FOV). This set, usually called a “phase vector”, can be integrated into the hardware as permanent or adjustable phase shifters implemented e.g in the form of coaxial cables of defined length. Alternatively, for the last generation of MR-scanners with support of pTX-mode (e.g. Magnetom™ “Terra", Siemens Healthineers), the default phase vector can be fixed in the coil configuration file to set the phases of driving voltages generated by RFPA. This provides operation of the mTX-array in the so-called “pTX Compatibility Mode”. The default phase vector should shape a reasonable combined *B*_*1*_^*+*^*-*profile for an average subject making possible a straightforward application of the array in the single-Tx mode without RF-shimming as well as simplifying the B_1_-shimming process for pTX mode. For the birdcage volume resonators or dipole antenna arrays with cylindrical symmetry of elements arrangement, this usually corresponds to the “circularly polarized” (“CP”) phases distribution based on the geometry of elements allocation [18]. However, the straightforward geometry-based approach for setting up default phases is not possible for the surface cardiac mTX-arrays having sophisticated shapes of individual elements and their allocation on a thorax. Therefore, for this type of mTX-arrays, the problem of default phasing is formulated as an optimization problem for a cost function maximizing homogeneity of the *B*_*1*_^*+*^*-*field with certain constraints or regularization on SAR and efficiency of using an available RF-power [5, 8, 11, 19, 20]. For typical numbers of Tx-elements (*N* = 8…16), the high-dimensional cost function has numerous local extremes. Many computational methods were applied in the context of both static and dynamic *B*_*1*_^*+*^ manipulations which include linear and non-linear solvers approaches. However, most of these methods are targeted for the application of the *B*_*1*_^*+*^-shimming during the scan session, to deliver practical solutions under a strong time limitation (~1 min computation time). Therefore in most cases, the result represents a local optimum dependent on starting point used for the search process initialization. Moreover, these methods are mostly developed for the *B*_*1*_^*+*^-shimming using pTX-RFPA and manipulating with both phases and magnitudes of the driving voltages. Due to this reason, the optimization techniques developed for the subject-specific *B*_*1*_^*+*^-shimming providing quick but locally optimal solution within limited FOV is suboptimal for the optimization of default array elements phases as a part of the coil development and final fine-tuning. In this case, the computation time could be extended to hours and the main goal is to achieve the globally optimal characteristics of a shaped B_1_^+^ in a frame of the formulated optimization problem.

For the small (N = 4–6) number of TX-elements, the most straightforward and universal solution of the discussed “phase only” optimization problem is the global brute force search over the complete phase space raster defined with reasonable discretization over each vector coordinate [21]. However, because the number of values in the raster grows exponentially with the number of elements this requires an extremely long computation time even for a moderate number of phase elements N≥8 and discretization step (e.g 5°). Therefore, often a prior experience and pragmatic restrictions on the searched phase vector components range are used to accelerate these computations. As an example, in the work [8] the brute force approach was used in a highly restricted phase space raster for 12 elements array (36^3^≈40000 vectors was used). This procedure was considered to be sufficient for a symmetrical rectilinear array geometry and suggesting that phases should not vary along the z-direction. Alternatively, in the work [6], the search of optimal phases was performed using a non-linear solver (NLS) approach with multiple random starting points (referred to further as “random multi-start”). However, for keeping computation time reasonably short, the number of starting vectors for the NLS-optimization was deliberately limited to 1000. For the mTX array with 16 elements, this number corresponds to ≈10^−24^ parts of the whole phase vector space gridded with 10° steps. As it’ll be shown further in this paper, in an arbitrary array elements configuration, the straightforward usage of random-multi-start strategy may not allow us to detect a sufficiently good approximation of a global optimum of the typical optimization cost functions. This paper aims to propose a flexible pragmatic approach for searching for an optimal phase setting to shape a targeted default *B*_*1*_^*+*^-field of mTX arrays. The proposed numerical optimization strategy allows for a flexible trade-off between (i) sufficiently short computation time and (ii) probability of reaching the global optimum of the targeted B_1_^+^ characteristics. It does not involve *a priori* constraints on the searched phase space (e.g. originating from the symmetry of the coil’s geometry). In the current work, the proposed technique was validated using an in-house developed 8TX/16RX dual part array for 7T cardiac MRI in pigs described in [22].

### Theory

The combined field created by an mTX array with the constant driving amplitudes is expressed as:

$$B_{1c}^{+}(r)=|\sum_{k=0}^{N}\Phi_{k}b_{1k}^{+}(r)|$$ (1)

Here, $\Phi_{k}=e^{i\varphi_{k}}$ are complex phasors of the driving current for channels and $b_{1k}^{+}(r)$ are spatial B_1_^+^-maps of the individual coil elements. The phasing of an array is performed by control of the phasor vector {**Φ**} = {Φ_1_⋯Φ_*N*_} to achieve the targeted spatial homogeneity of combined field *B*_*1c*_^*+*^*(r)*.

#### Cost functions and optimization problem

In general, the goal of *B*_*1*_^*+*^-shimming is to achieve a uniform excitation pulse flip-angle (FA) distribution within а whole imaged FOV. However, using static $B_{1}^{+}‐\mathrm{shimming}$ and manipulating only with phases of individual elements this task would be either non-accomplishable or put very strong limitations on the range of achievable FA. Therefore, a pragmatic approach demands a certain degree of $B_{1c}^{+}$ homogeneity characterized by statistical metrics of a distribution uniformity to be reached in the limited region-of-interest (ROI) *Δr = (*Δr_x_,Δr_y,_Δr_z_*)*.

One of the widely used uniformity metrics used for the B_1_^+^-field shimming is the coefficient of variation:

$$CoV(\Delta r)=\frac{std(B_{1}^{+}(\Delta r))}{mean(B_{1}^{+}(\Delta r))}$$ (2)

Here *mean()* and *std()* denote the mean value and the standard deviation calculated over the voxels within Δ*r*. Similar to the work [23] we introduce a regularization factor Δ_*m*_ to enhance the demand on homogeneity and compose the spatial uniformity cost function *F*_*u*_ as:

$$F_{u}(\Delta r,\{\Phi \})=\frac{1}{CoV(\Delta r)∙\Delta_{m}(\Delta r)};\;\Delta_{m}(\Delta r)=\frac{\max\left({B_{1c}}^{+}(\Delta r)\right)-mean({B_{1c}}^{+}(\Delta r))}{mean({B_{1c}}^{+}(\Delta r))}$$ (3)

Among an achievable B_1_^+^-homogeneity, the essential characteristic of an mTX-array is the efficient usage of the available radiofrequency power. This can be controlled in the optimization procedure via an array transmit efficiency ratio of “sum-of-magnitudes” (SOM) and “magnitude-of-sum” (MOS) determined as [18, 21, 23]:

$${TX}_{e}(\Delta r,\{\Phi \})=\frac{\left|\sum_{k}^{N}\Phi_{k}s_{k}^{+}\right|}{\sum_{k}^{N}\left|\Phi_{k}s_{k}^{+}\right|}$$ (4)

Taking values in the range [0… 1] it characterizes the efficiency of power usage at the specific combination of phases Φ_k_ producing $B_{1c}^{+}$.

Finally, the optimization problem for the combined cost function *F*_*c*_ to be solved for the determining phasor vector can be formulated as follows:

$$\{\Phi_{\mathrm{opt}}\}=\mathrm{argmax}\left(F_{c}(\Delta r,\{\Phi \})\right)\;F_{c}(\Delta r,\{\Phi \})=F_{u}(\Delta r,\{\Phi \})∙{TX}_{e}(\Delta r,\{\Phi \})$$ (5)

## Method

### Computation over sub-sampled phase space

The essential difficulty of solving the problem (5) is the periodic influence of each component of the phasor vector *Φ*_*k*_ on the cost function *F*_*c*_ leading to multiple local extremes (S1 Fig). In practice, this means that an optimization search initiated at a specific point *{****Φ***_***0***_*}* ends up in the nearest local extremum *{****Φ***_opt_*}*. This often makes a straightforward application of both derivative-based and other types of local NLS inefficient.

The most straightforward and universal solution for the problem is using a global *exhaustive search* varying all possible combinations of phasor vector components with a discrete step. However, for the given step δΦ and *N* Tx-elements, the global full-exhaustive search requires computing the cost function (360/δΦ)^N^ times which for the δΦ≈10° and N = 16 would lead to more than 10^25^ multiplications of complex matrices of size (Δr_x_·Δr_y_·Δr_z_). The estimated computation time (up to several months on a high-performance workstation with multicores CPU) makes such an approach technically non-practical. In this work, we propose the computation strategy with an intelligent fusion of the brute-force approach and usage of local solvers to find a sufficiently good approximation of the global maximum of the cost function and, thus, globally optimal combined *B*_*1*_^*+*^-profile.

**Fig 1:**
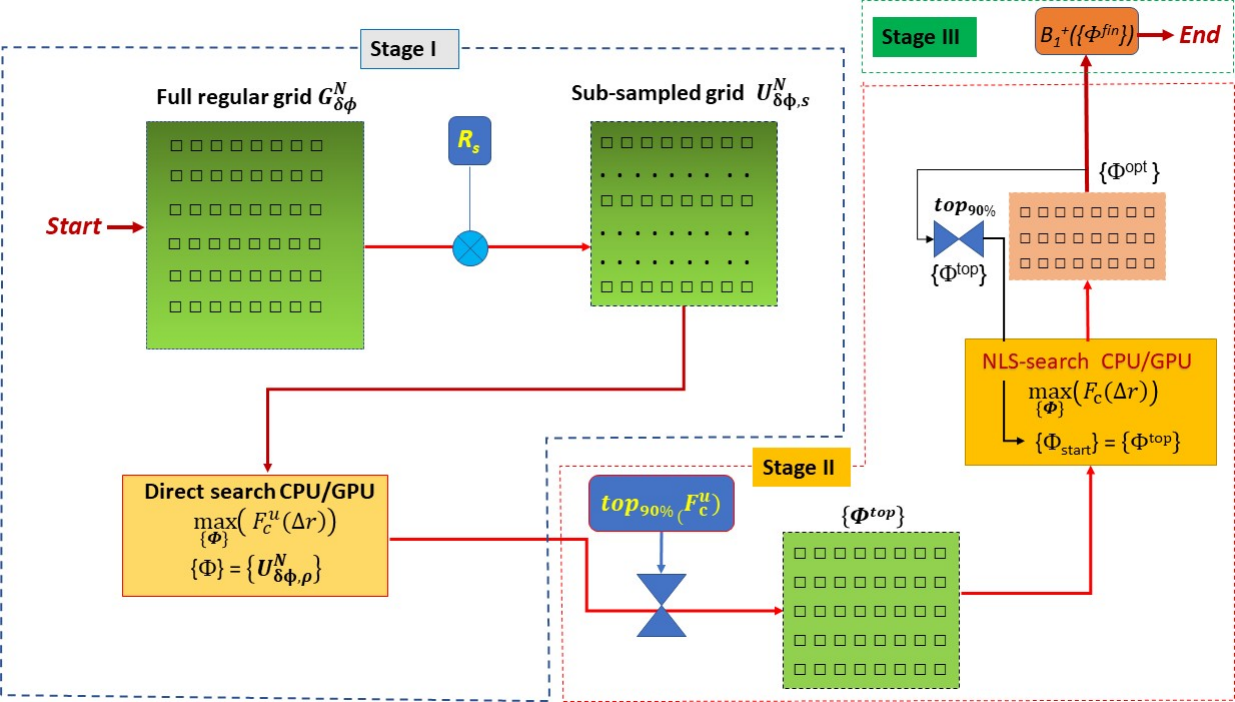
Flowchart of the phase optimization algorithm. At stage II, phase vectors {Φ^top^} providing cost function values F_c_ above 90% of maximum are selected as initial condition {Φ_0_} for iterative NLS-search and selection of the optimum vectors {Φ^opt^}. The vector sets found by this algorithm undergo final selection by (additional) user-defined criteria in Stage III to provide final set(s) {Φ^fin^}.

The complete flowchart scheme of the proposed computation method is shown in Fig 1. The goal of the first stage (Stage I) is to find a reasonable finite discrete representation of *F*_*c*_*(Δr*_,_{**Φ**}*)* using the *N*-dimensional discrete phase space {**Φ**}. For this purpose, the discrete grid of phase vector with N components should be formed as

$$G_{\delta \phi }^{N}\;with\;{\left\{\phi_{i}\right\}}_{L}=\{\phi_{i}^{0}\pm \delta \phi \ldots \phi_{i}^{0}\pm L∙\delta \phi \}$$ [6]

providing the “full-grid” discrete form of the cost function $F_{c}(G_{\delta \phi }^{N})$, where *L* denotes the total number of nodes in the grid.

The further approach relies on two observations: (i) the cost function *F*_*c*_({Φ}) is periodic by each phase vector component and (ii) the set of values {Φ^*top*^} providing values above 90% of its top *(F*_*c*_{Φ^*top*^} *>0*.*9*·*max(F*_*c*_*))* is formed by relatively tiny (<10^−6^) part of all vectors in full grid $G_{\delta \phi }^{N}$ (see S1 Fig). Therefore, as the second step, the $G_{\delta \phi }^{N}$ is randomly sub-sampled by the sampling operator *R*_*s*_ with the uniform probability density function (PDF). This operation selects *L*_*s*_ = *L*^*N*^/*s* vectors from $G_{\delta \phi }^{N}$, where *s* denotes the scaling factor of the effective nodes number reduction. In this work, the sub-sampling was performed using the uniform PDF. The usage of alternative sampling schemes (e.g. with Poisson disk PDF) for a further improvement of the computation efficiency of the proposed method can be also considered. As the result, this creates a “sub-sampled” grid:

$$U_{\delta \phi ,s}^{N}=R_{s}[G_{\delta \phi }^{N}]$$ (7)

The phasor vector sub-space with the reasonably chosen subsampling by factor s≈10^3^ for N = 8 can be used to find the great majority of the summit points of the cost function $F_{c}^{u}=F_{c}(U_{\delta \phi }^{N})$ within practically usable computation time on the middle-class CPU or GPU.

Further, in Stage II, the vectors {Φ^*top*^} such as $F_{c}^{u}(\{\Phi^{top}\})>0.9\cdot max(F_{c}^{u})$ are used as starting points in the NLS-optimization of the problem (5) to find phasor vector {Φ^*opt*^}. The number of vector *N*_*top*_ forming {Φ^*top*^} typically varies in the range of several dozens depending on the under-sampling factor *s* and can be reduced if necessary by setting up a higher threshold for selecting the summit values (for example $F_{c}^{u}(\{\Phi^{top}\})>0.975\cdot max(F_{c}^{u})$). Depending on starting point and volume of the optimization region it requires NI≈5-10·10^3^ optimization solver iterations to find the local optimum of the cost function for a single starting point in {Φ^*top*^}. This procedure is repeated iteratively (see Fig 1) to reduce the set of phasors {Φ^*opt*^} to a sufficiently small amount (N<10) which can be considered as a good approximation of the global optimum. The *B*_*1c*_^*+*^ field shaped by these vectors {Φ^*fin*^} can be analyzed based on various preference criteria for (e.g. minimal *B*_*1c*_^*+*^ gradients, maximal peak FA, best possible slice profile, etc) to select the variant used for the array default setup. In this work Stage II was performed using “*fminsearch”* Matlab function implementing the Nelder-Mead algorithm of optimization and known as less dependent from the starting point in comparison to other local solvers [24]. The performed in the Stage I preselection of the starting points allows for starting NLS-search close to the summits of the cost function and, thus, essentially increases the probability that the found solution vector will be globally optimal for the problem (5).

### B_1_^+^-profiles of the transmit elements

The described *B*_*1*_^*+*^ optimization strategy relies on the knowledge of the complex B_1_^+^-maps of each Tx-element *b*_*1k*_*(r)*. However, to acquire the experimental B_1_^+^-maps a fully functional prototype of the array is required. Moreover, measuring these maps experimentally with sufficient reconstruction quality is often a non-trivial problem. The destructive interferences lead to attenuation of the MR-signal and producing artifacts of reconstruction or voids in the B_1_^+^-maps. If this takes place in the region where B_1_^+^-shimming is targeted, the reliable optimization becomes problematic or requires significant efforts for data curation.

The alternative efficient approach is to use numeric electromagnetic simulations to compute the 3D distribution of the electromagnetic fields created by individual coil elements. After validating the simulated data by the experimental MR-measurements it can be further employed for the computation of the combined *B*_*1c*_^*+*^ in any arbitrarily placed region of interest. Moreover, this allows to test the capabilities of the specific array design in terms of tailoring desired B_1_^+^ profiles “in-silico” and perform necessary optimizations of both physical elements arrangement and electrical circuits [25].

In this work, the electromagnetic fields of the mTX-arrays were calculated using CST Studio Suite (3DS, Dassault Systems). The time-domain solver was used for the EM-simulation of the array’s structures and the whole phantom volume within the coil using 4·10^7^ mesh cells. The average computation time using a dual-core Intel Xeon^(TM)^ E2650 CPU and two NVIDIA Tesla^(TM)^ K80 GPUs was 48 hours for each simulated structure. The simulated data have been exported as 3D complex H-field values with the isotropic spatial resolution voxels of 4x4x4mm. Further computations were performed using in-house developed Matlab scripts (Matlab 2017a/b, Mathworks, USA).

### Testing and validation of the phase space under-sampling strategy

To test and validate the efficiency of Stage I strategy in terms of a sub-sampling factor the optimization of phase vector for the six paired elements (Tx1-Tx4, Tx6, Tx7) was performed. These elements provide ~90% of the total contribution in the combined *B*_*1*_^*+*^ field in the center of the ROI targeted for the testing. Reducing dimension made it possible to perform computation over a full phase space grid and grid undersampled by low factors *s* within a reasonable time. The full phase space grid *G*_*L*_ was built according to [6] using *L* = 17 nodes. With the span of {ϕ_k_} components within [-180°..180°] it provides the discretization step *δϕ* ≈ 20^0^. The full grid was sampled according to [7] to create the sub-sampled grids *U*_*s*_ with densities varying in a range of s = [1..10^4^]. The grids with the different sub-sampling factors were used to test the fidelity of approximation of the cost function *F*_*c*_ by the sub-sampled version $F_{c}^{u}$. This was done by comparison of the maximal values *F*_*c*_ and $F_{c}^{u}$. These values were computed for the varied size of regions *Δr* and values of sub-sampling factor *s* to test if the sufficient amount of top values of the “full-grid” cost function *F*_*c*_ are preserved in the sub-sampled $F_{c}^{u}$. The profiles of $F_{c,top}^{u}(s)/F_{c,top}$ at different values were analyzed.

### Usage of CPU and GPU

In general, the efficiency of GPU computations depends on the GPU performance index and dimensionality of the problem, and the size of processed arrays. For N = 8 elements no advantage of GPU usage was found. However, the situation changes significantly for a higher-dimensional problem with N = 16 elements optimization. For the CPU the computation time grows roughly proportional to the number of voxels in the optimized ROI. At the same time for the GPU, the computation rate for both Stage I and Stage II calculations remains practically independent of the size of computed arrays up to 2.5·10^4^ voxels in the optimized ROI (corresponds to 12x12x12cm volume). S2 Fig shows that computation time with a modern GPU may become drastically shorter compared to the CPU already starting from a targeted volume of ~1300 voxels. Therefore, in practice, the decision of usage of GPUs within the computation pipeline should be based on benchmarking of small portions of the actual phase grid *U*_*s*_ (e.g ~0.5%) for the particular dimensionality of the problem and optimization volume.

### Comparison to a traditional optimal phase computation method

To analyze the improvement of the homogeneity and mean value of B_1_^+^ which can be obtained with our optimization method we compared it to the random multi-start optimization [6]. For *N = 8* optimization a randomly selected set of 1000 phasor seed values with uniform distribution of components *φ*_*k*_ in the range of [-180°..180°] was used as a starting point. The NLS-search was performed using the function (5). To provide sufficient statistics for the NLS-search comparison, the number of initial phasors {Φ_0_} for the pre-optimized start search was set to be 250. This corresponded to a cost function cut-off level of ≈70% from the top (the cut-off at 90% typically provides ~20–30 phasors for Stage II). The comparison of the optimization methods was performed for an ROI within a spherical phantom P_1_ (described below). The ROI with dimensions Δr = 50x50x50mm was placed as shown in Fig 5. Because the major intrinsic *B*_*1*_^*+*^ inhomogeneity for the surface array is the gradient in the anterior-posterior (“Y-axis”) direction, the optimized *B*_*1c*_^+^ profiles were compared using the relative standard deviation of the mean intensity projection computed as $\vartheta_{r}=std(b_{1m}^{+})/<b_{1m}^{+}>$ and its relative absolute gradient $|{\nabla b_{1m}^{+}|}_{r}=|\nabla b_{1m}^{+}|/<b_{1m}^{+}>$. These projections were computed in z-direction for the slab Δz = 50mm.

As the final step, the comparison of an optimized and random multi-start was done for a high dimensional problem (*N* = 16 elements). The optimal phase vector search was done using the simulated elements *B*_*1*_^*+*^*-*maps of the array loaded by the numerical phantom P_2_ (described below). The dependence of the achieved optimization result from the number of starting phasors *N*_*start*_ was analyzed using 3 repetitions of NLS-optimization with *N*_*start*_ *=* 7500. The cumulative maximum of the cost function was computed as max ($F_{c}^{opt}$ [*1*..*N*_*start*_]), where *N*_*start*_ increments from 1 to 7500.

### Coils, phantoms and MRI validation of electromagnetic simulation data

The mTX-array used for the validation consists of two physically independent parts each comprised of eight loop elements. The array is connected to the scanner via a dedicated RF-interface (Rapid Biomedical, Rimpar, Germany) which allows for using connected coils both in sTx and pTX modes. A detailed description of the array design and hardware specifications can be found in [22]. The driving voltage phases of elements can be adjusted in two ways:

1. Individually for each of 16 elements using phase-shifting coaxial cables connected to the dedicated sockets of the RF-interface.
2. Pairwise for every two elements, using 8 RFPA channels available on the scanner in pTX regime.

**Fig 2:**
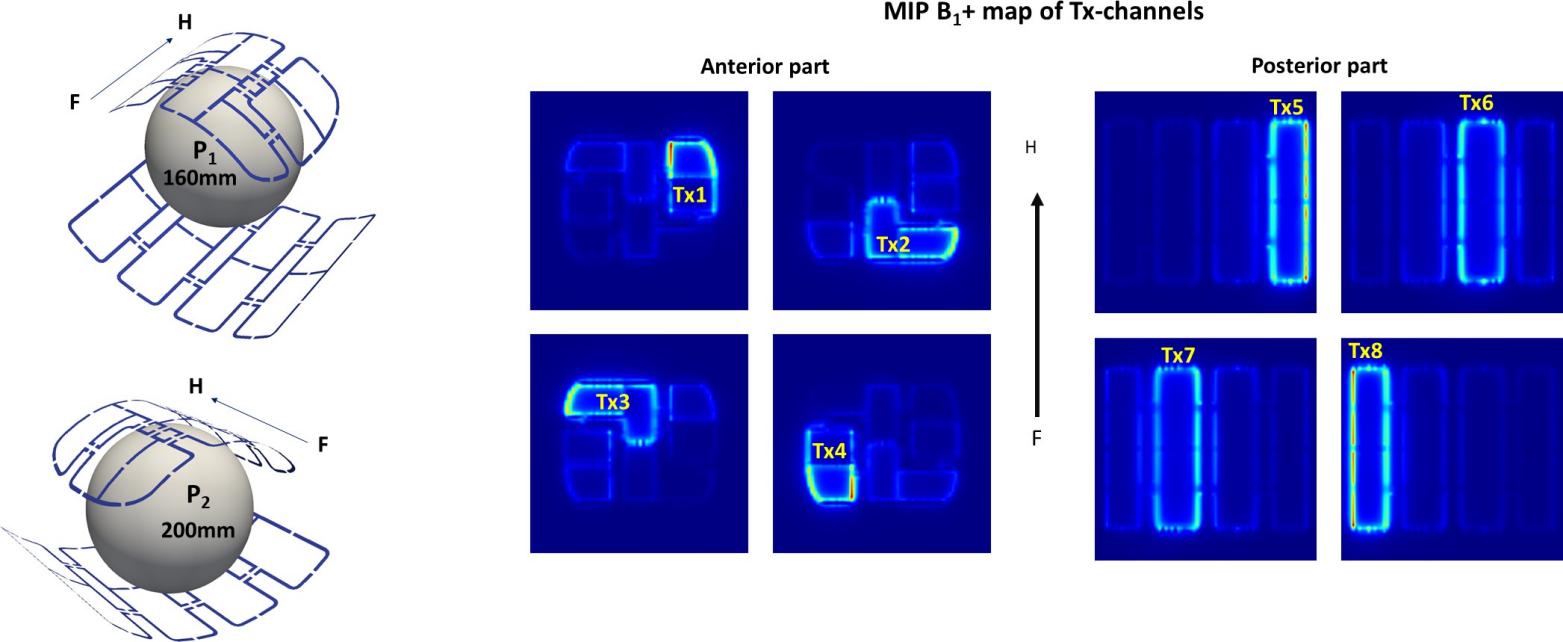
(a) Experimental 8TX/16RX array with antisymmetric L-shaped elements used for demonstration of the described phase optimization method efficiency. The position of the 160mm (P_1_) and 200mm (P_2_) spherical phantoms used in both computation and MRI-measurement are shown. Panel (b) shows the coil elements of both parts (top array, bottom array) on the maximal intensity projection images (MIP) of the CST-computed B_1_^+^ field.

Fig 2 shows a sketch of the array elements’ configuration and location in both anterior and posterior parts along with the elements pairing scheme for driving by 8TX channels in pTX regime. Two phantoms we used for testing the proposed method both in the numerical simulations and in the experimental validation. Phantom P_1_ comprised six rectangular cross-section PE bottles loading the posterior part, and a plexiglass sphere (diameter, 160mm) positioned inside the anterior part (Fig 2 left panel). The bottles and the sphere P_1_ were filled with a solution of sugar and NaCl mixed in an experimentally defined proportion providing permittivity close to the typical muscle tissue (ε≈58). The second phantom P_2_ represents an acryl glass sphere with a diameter of 200mm filled with PVP solution prepared as described in [22] and [26].

The spherical phantom was chosen based on practical experience to minimize the number of destructive RF-interferences within the ROIs corresponding to the typical position of an animal’s heart relative to the array. Simultaneously, this mimics real animal studies where some of the anterior array elements are more distant from the loading tissue than others.

All MRI measurements were performed using a 7T Magnetom^(TM)^ Terra whole body 7T scanner (Siemens Healthineers, Erlangen, Germany) equipped with an 8TX channels RFPA. The FA measurement for the *B*_*1*_^*+*^*-*map reconstruction was done with the double angle mapping (DAM) technique based on a GRE sequence [27]. Additionally, the *B*_*1*_^*+*^*-*maps were cross-validated with the vendor’s *B*_*1*_^*+*^*-*mapping pulse sequence (turbo-FLASH with magnetization preparation [28]) available on the scanner. For the GRE-DAM measurements, the parameters were: TE/TR = 1.8/4000ms, pixel resolution 2mm^3^, slice thickness 4mm. The sequence parameters for the Turbo-FLASH *B*_*1*_^*+*^*-*mapping were TE/TR = 1.7/9000ms, FA = 10^0^ for the same spatial resolution. The coronal slice providing the best overview of the Tx-profiles of the anterior array elements was chosen for comparison with the CST-simulated maps. The FA-map reconstruction was performed using an in-house developed Matlab script. The agreement of simulated and measured data was checked numerically by linear regression between normalized 1D-profiles computed along the lines crossing the most prominent features of simulated and measured 2D FA-maps in each channel.

Testing the efficiency of the computed phases for *B*_*1*_^*+*^ optimization was done using the same GRE-sequence as for DAM measurements. The initial “zero” phasеs of the array were set by manual adjustment geometrically guessed phase distribution [22]. To visualize the effect of *B*_*1*_^*+*^ optimization the volumes inside the iso-surface SNR>30 were compared using 3D stack of GRE images. SNR was computed on pixel bases as *SNR(x*,*y*,*z) = S*_*i*_*(x*,*y*,*z)/σ*_*m*_. The *S*_*i*_ denotes signal intensity in pixel and *σ*_*m*_ is the mean standard deviation of the intensity of the pixels in the “noise pool” represented by 20x20 pixel regions in each corner of the FOV for all acquired slices.

## Results

Fig 2A demonstrates the schematic of array elements and the position of the spherical phantom P_1_ in EM-simulations and MRI-measurements. Both panels of Fig 2B show the maximal intensity projection (MIP) of simulated magnitudes of B_1_^+^-fields for each element pair combined to be driven by each of eight Tx-channel (labeled accordingly). The enhanced lines on MIP of B_1_^+^-maps show the position of the element’s loops.

**Fig 3:**
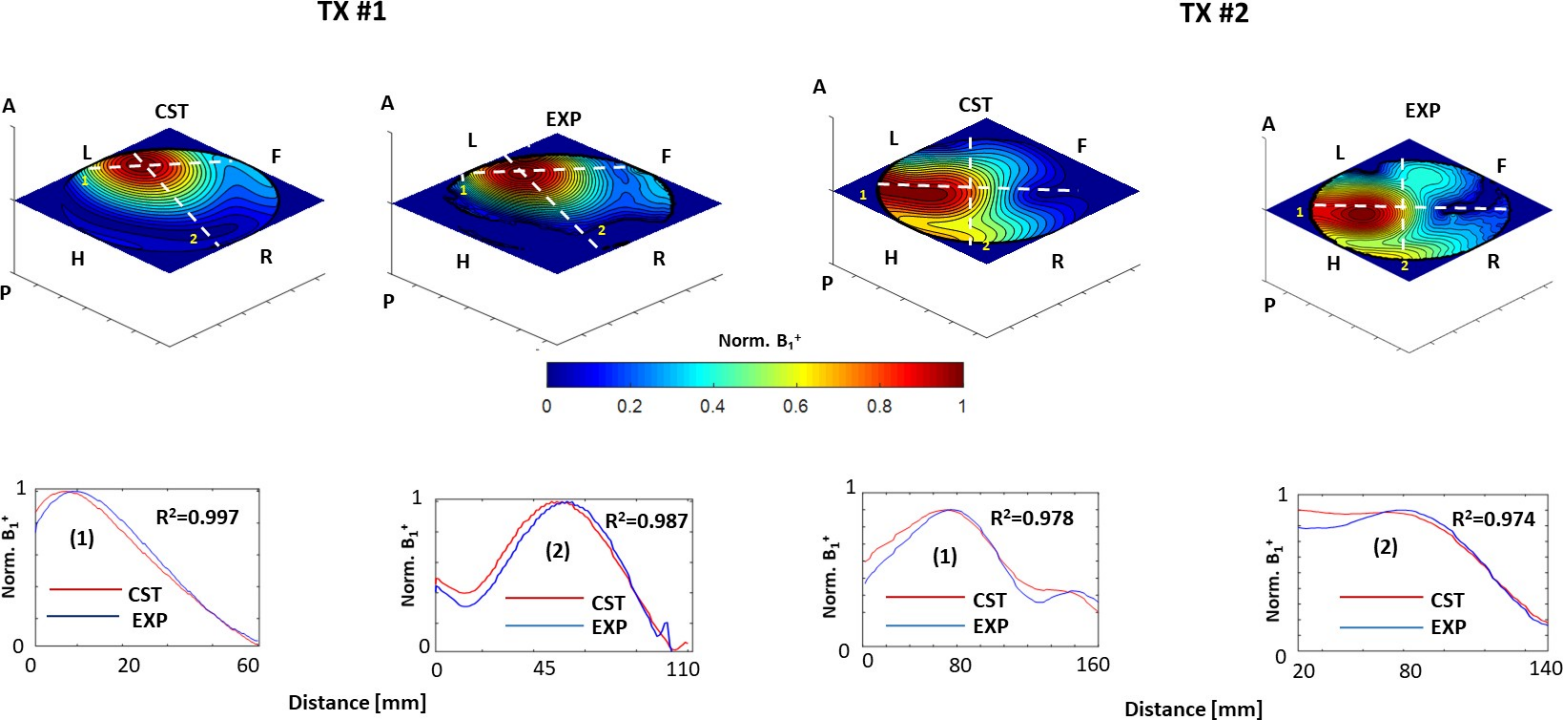
Comparison of simulated in CST (“CST”-label) and experimentally measured (“EXP”-label) B1+ fields of pig cardiac array using phantom P_1_. Central coronal slice B_1_^+^-maps are simulated and measured in the spherical phantom. Labels A, P, H, F, L, R denotes corresponding spatial directions in the scanner coordinate system. Two normalized maps of individual Tx-channels of the array are shown as an example. The top row shows good agreement of 2D maps (in particular, positions of maxima and minima). The bottom row demonstrates 1D profiles (1) and (2) extracted along directions marked by dashed lines on 2D maps. The close agreement of simulated and measured profiles is confirmed by high coefficient of determination of linear regression between both data.

Fig 3A and 3B demonstrates exemplary results from the validation of the CST-computed *B*_*1*_^*+*^ maps for individual Tx-channels using MRI measurements. The plotted contour diagrams demonstrate a very good qualitative agreement in the 2D spatial distribution of the *B*_*1*_^*+*^-field between experiment and simulation. Additionally, the 1D profiles computed across characteristic features of the B_1_^+^-maps show good quantitative agreement. The computed coefficients of determination of the linear regressions between experimental and measured profiles are in the range *R*^*2*^ = [0.97–0.99]. This confirms the possibility of using the CST-simulated *B*_*1*_^*+*^ profiles for the optimization of the combined *B*_*1c*_^*+*^. The comprehensive validation of the array CST model with full 2D channel-by-channel *B*_*1*_^*+*^ maps can be found in

**Fig 4:**
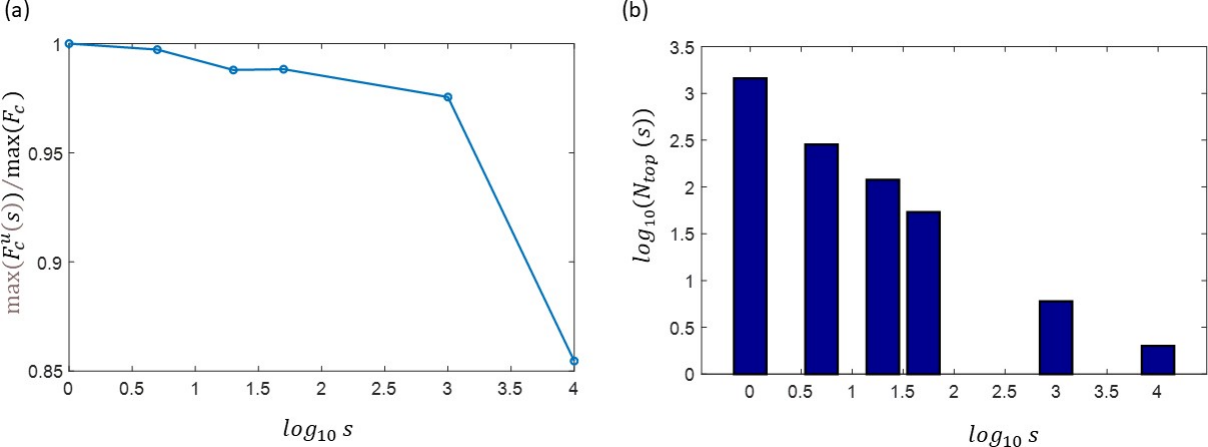
Detection of the top decile of the cost function *F*_*c*_ using sub-sampled phase space *U*_*s*_. Panel **(a)** shows the value $max\;(F_{c}^{u})/{max\;(F}_{c})$ computed using *U*_*s*_-grids with sub-sampling varied in the range of *s* = [1– 10^4^]. Panel **(b)** demonstrates that the number of the vectors *N*_*top*_ selected by Stage I is fairly inverse proportional to the size of the searched grid (defined by the factor *s*).

Fig 4A demonstrates the results of testing the capability of using in the search of the top values a sub-sampled cost function $F_{c}^{u}$ to capture the summit points of the fully sampled function *F*_*c*_. Plot 4a shows ${max\;(F}_{c}^{u})/{max\;(F}_{c})$ values computed using *U*_*s*_-spaces with sub-sampling factor in the range of *s* = [1– 10^4^]. One can see that for *s<10*^*3*^ the values of ${max\;(F}_{c}^{u})/{max\;(F}_{c})$ are very close to 1. The value *N*_*top*_ denotes the number of vectors in the set{Φ_*top*_} forming the summit of $F_{c}^{u}$, such as $\frac{F_{c}^{u}\{{}_{top}\}}{\max\left(F_{c}^{u}\right)}>0.9$. This number reduces proportional to the sub-sampling factor (Fig 4B). That way, the preselection of vectors from a space $U_{\delta \phi ,s}^{N}$ performed in the Stage I should provide the same set of optimal starting points for the NLS-optimization as if a full grid $G_{L}^{N}$ would be used. Therefore, choosing sub-sampling factor *s* in the range *10*^*2*^..*10*^*4*^ should provide the set of vectors {Φ_*top*_} which due to the sufficient proximity to the global maximum of the cost function will ensure high efficiency of the NLS-based optimization in Stage II.

**Fig 5:**
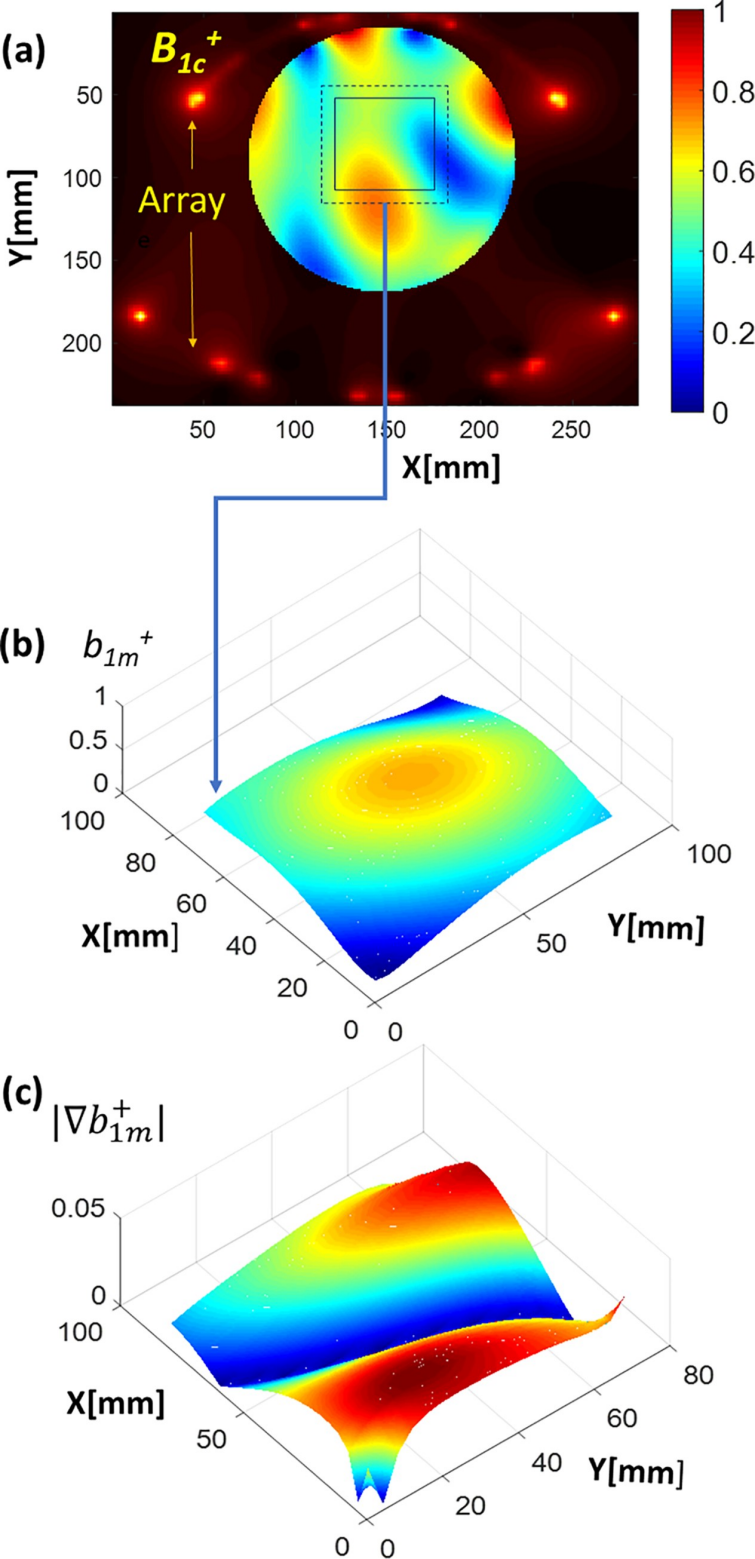
Panel **(a)**. The solid square marks the position (xy-projection) of the targeted optimization region within the spherical phantom. The region is used to compare two optimization methods efficiently. Panel **(b)** shows the mean intensity projection of *B*_*1c*_^*+*^ through z-direction computed *before optimization* (denoted as *b*_*1m*_^*+*^). The zoomed target region labeled with a dashed line on panel **(a**) is plotted. Panel **(c)** shows the absolute gradient of the *b*_*1m*._^*+*^. An essential inhomogeneity of *b*_*1m*._^*+*^ before the optimization is observed.

Fig 5A demonstrates CST-simulated combined B_1_^+^-map in the spherical phantom P_1_. Маp were computed with a default zero phasor {*φ*_*k*_} = 0. The marked square ROI was selected for the comparison of the standard (random-multi-start) and proposed novel optimization approaches. Fig 5B shows the B_1_^+^ field and the absolute value of its gradient demonstrating the existing B_1_-inhomogeneity prior to the optimization of the elements phases.

**Fig 6:**
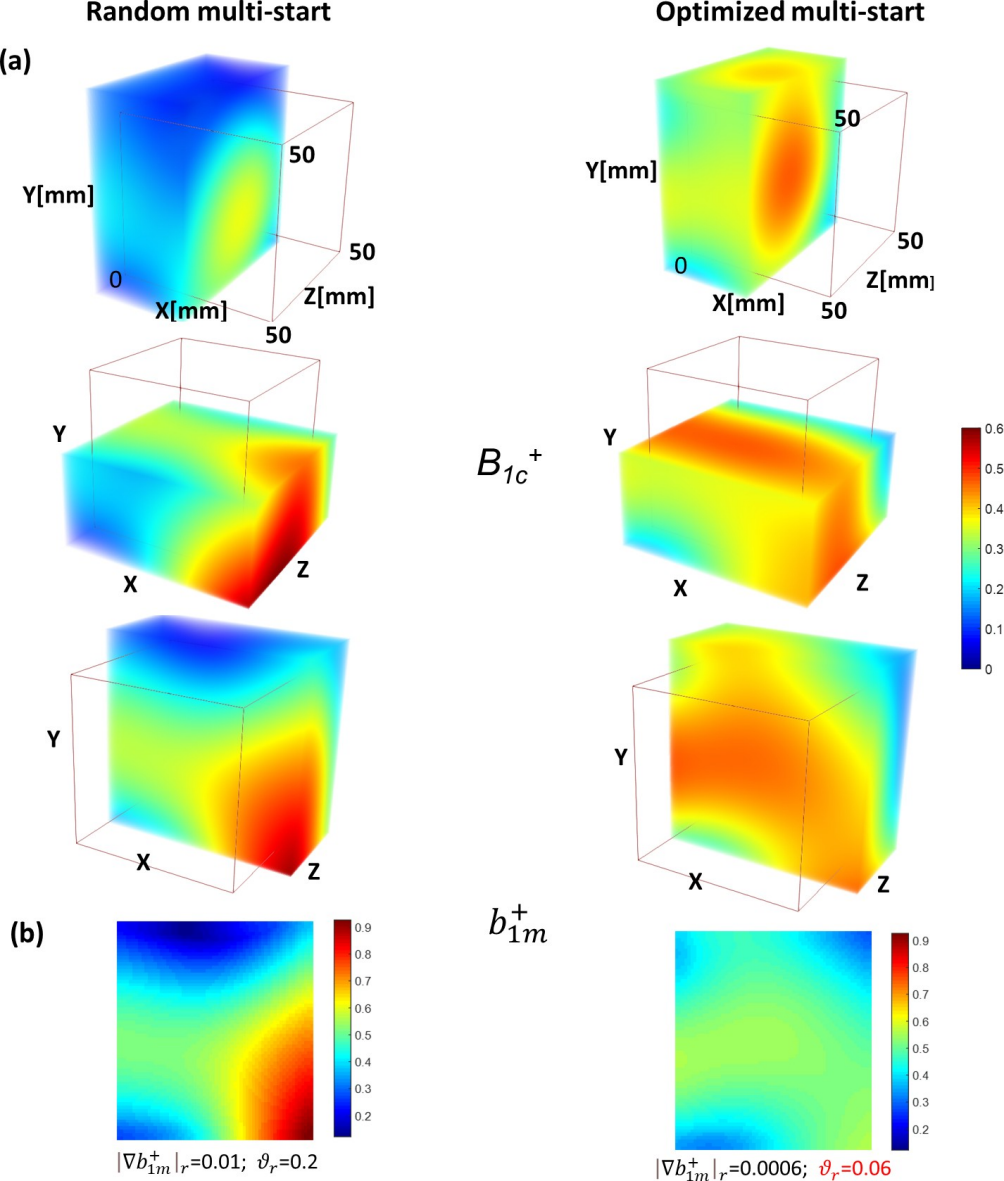
Comparison of the new phase-optimization methodology (“optimized multi-start”) with the conventional one (“random multi-start”). Top panel (a) shows the 3D rendering of 50mm thick volumes of interest in different orientations. Panel (b) presents a mean intensity projection through the z-axis direction of the resulting B_1c_^+^. The optimized starting points of the phase search result in a significantly higher homogeneity of the B_1c_^+^-field.

Fig 6 depicts the results of the optimization of the phases for N = 8 paired elements performed with the standard random-multi-start approach and the proposed novel dual-stage method of phase optimization. Panel (a) demonstrates volume-rendered *B*_*1c*_^*+*^ cuts at the center of the optimization volume. Panel (b) shows the mean intensity projection of *b*_*1m*_ over z-dimension within the 50mm slab. The optimized dual-stage multi-start approach provides better”focusing” of the homogeneous area of the *B*_*1c*_^*+*^ within the optimized volume in comparison to the random multi-start. The improved homogeneity metrics of *<b*_*1m*_*>* achieved with the dual-stage optimized multi-start method confirm the optical impression.

**Fig 7:**
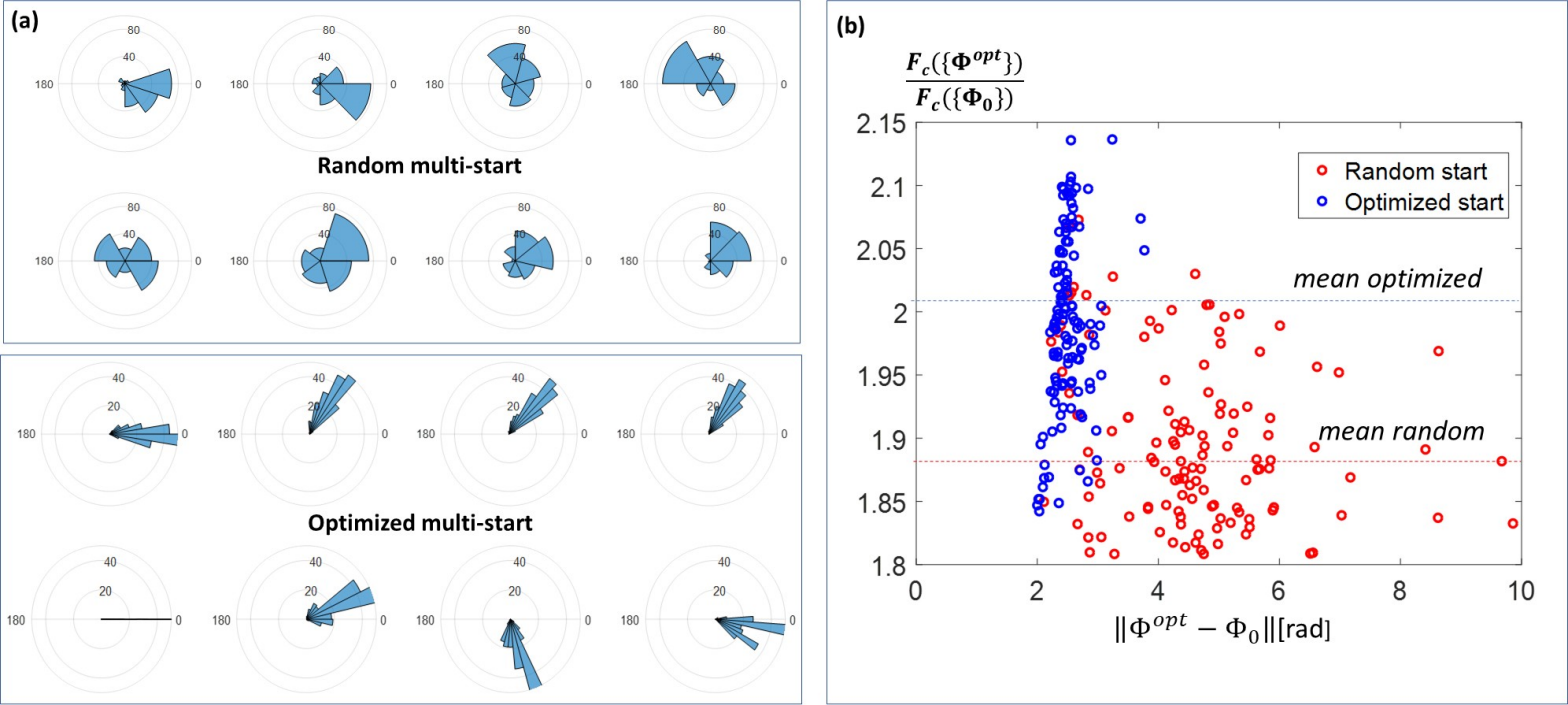
Panel (a) shows the distribution of the optimized phasor components obtained by the optimized and random multi-start. The standard deviation does not exceed 20^0^ (top panel) for the optimized multi-start. At the same time, the NLS-optimization with random starting points (bottom panel) gives rise to distribution with the spread reaching 180^0^. Panel (b) shows the dependence of the cost function grow-up rate *F*_*c*_({*Φ*^*opt*^})/*F*_*c*_({*Φ*_0_}) from the norm of the difference of the starting and an optimal phasor vector ‖Φ^*opt*^−Φ_0_‖.

Fig 7A demonstrates a difference in the distribution of the final optimized phasor components found by the NLS optimization using (i) the pre-optimized starting vectors selected by Stage I and (ii) random starting vectors with uniformly distributed components. In both cases, the angular histograms for each phase component were built using the 250 phasors which provided the largest values of the cost function. One can see that pre-optimized phase vectors used as starting points for NLS-optimization provide very high coherence of the final resulted vectors. The individual components of the phasors are spread in relatively narrow angular sectors with the standard deviation not exceeding 20^0^ (top panel). At the same time, the NLS-optimization with random starting points (bottom panel) results in the spread of phasor’s components in the range of up to 180^0^.

Fig 7B shows the dependence of the cost function progression ratio in the optimization process, defined as *F*_*c*_({*Φ*^*opt*^})/*F*_*c*_({*Φ*_0_}), from the norm of the initial and the final phasor vector difference *δ*Φ^*opt*^ = ‖Φ^*opt*^−Φ_0_‖. One can see that the length of the “search trajectory” *δ*Φ^*opt*^ is nearly constant (≈2 rad) for the pre-optimized starting phasors. At the same time, for the random starting phasors one observes a widely spread distribution of *δ*Φ^*opt*^ in the range of 2 to 10 rad. The median value of the cost function grow-up rate performed with the pre-optimized starting phasors is ≈25% higher than that of random starting points (marked by dashed lines of corresponding colors).

**Fig 8:**
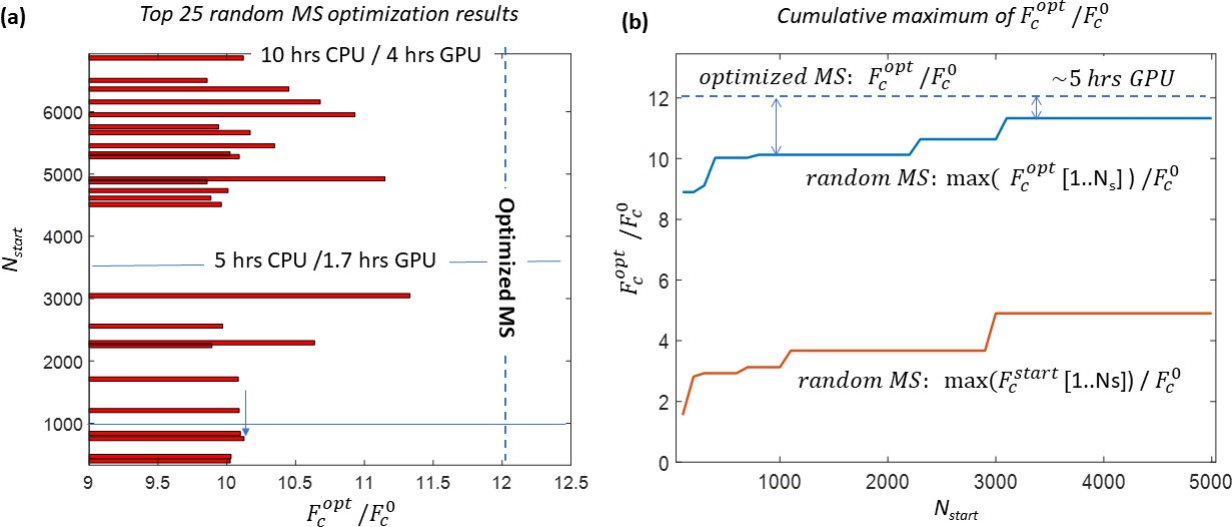
Efficiency of the random multi-start NLS-optimization for high dimensional (N = 16) problem using 7500 random starting phasors. Panel (a) shows the index of starting vector for the 25 top final values of the normalized optimized cost function $F_{c}^{opt}/F_{c}^{0}$. The approximate timings of the computations for ~50% and 100% of starting random vectors set are shown. Panel (b) shows the cumulative maximum of the normalized starting and optimized cost functions $F_{c}^{start}/F_{c}^{0}$ and $F_{c}^{opt}/F_{c}^{0}$ depending on the starting vector index (red and blue lines respectively). The dashed blue line shows the level of the same value achieved using optimized multi-start with ~5·10^7^ vectors in Stage I.

Fig 8 demonstrates the efficiency of the random multi-start NLS-optimization for high dimensional (N = 16) problem (5) performed with 7500 starting vectors. Panel (a) shows the index of starting vector for the 25 highest final values of the cost function achieved by 3 repetitions of random multi-start NLS-optimization. For the convenience of comparison, the final function $F_{c}^{opt}$ is normalized to the value at zero phase vector $F_{c}(\left\{0\right\})=F_{c}^{0}$. The approximate timings of the computations for ~50% and full starting random vectors set are shown. The cost function value achieved with the optimized multi-start within comparable (~5hrs on GPU) computation time is shown. Panel (b) shows the cumulative value of the maximum of the normalized optimized cost function $F_{c}^{opt}/F_{c}^{0}$ depending on the starting vector index. The dynamic of the reached optimized value demonstrates that optimization with a random multi-start for a high dimensional optimization problem (N = 16) requires the number of starting vectors essentially exceeding 1000.

**Fig 9:**
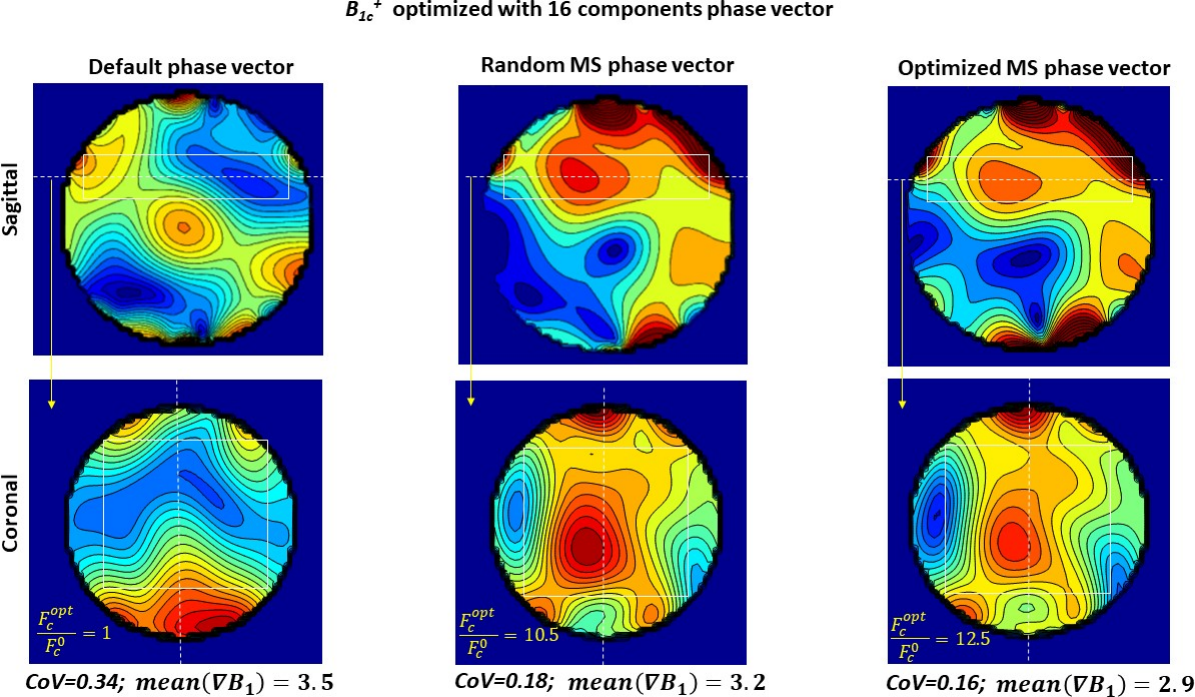
**Combined B**_**1c**_^**+**^ **in the targeted region (marked by the white lines) shaped with zero phase vector (left) and vectors found by random (middle) and optimized multi-start (right) optimization.** Sagittal and coronal slices at positions respectively marked with dashed white lines are shown. The value of the normalized cost function $F_{c}^{opt}/F_{c}^{0}$, coefficient of variation and mean gradient demonstrate an improvement of B_1c_^+^ homogeneity achieved by the proposed method.

Fig 9 demonstrates the results of optimization of the combined B_1c_^+^ shaped by N = 16 individual array elements in the marked white rectangle region of the phantom P_2_. The B_1c_^+^-field in the central sagittal and coronal slices of the optimized region for zero phase vector (left) and phase vectors found by random (middle) and proposed optimized multi-start optimization (right) are shown. The value of the normalized cost function $F_{c}^{opt}/F_{c}^{0}$, coefficient of variation and mean gradient demonstrate an improvement of B_1c_^+^ homogeneity achieved by the proposed method.

**Fig 10:**
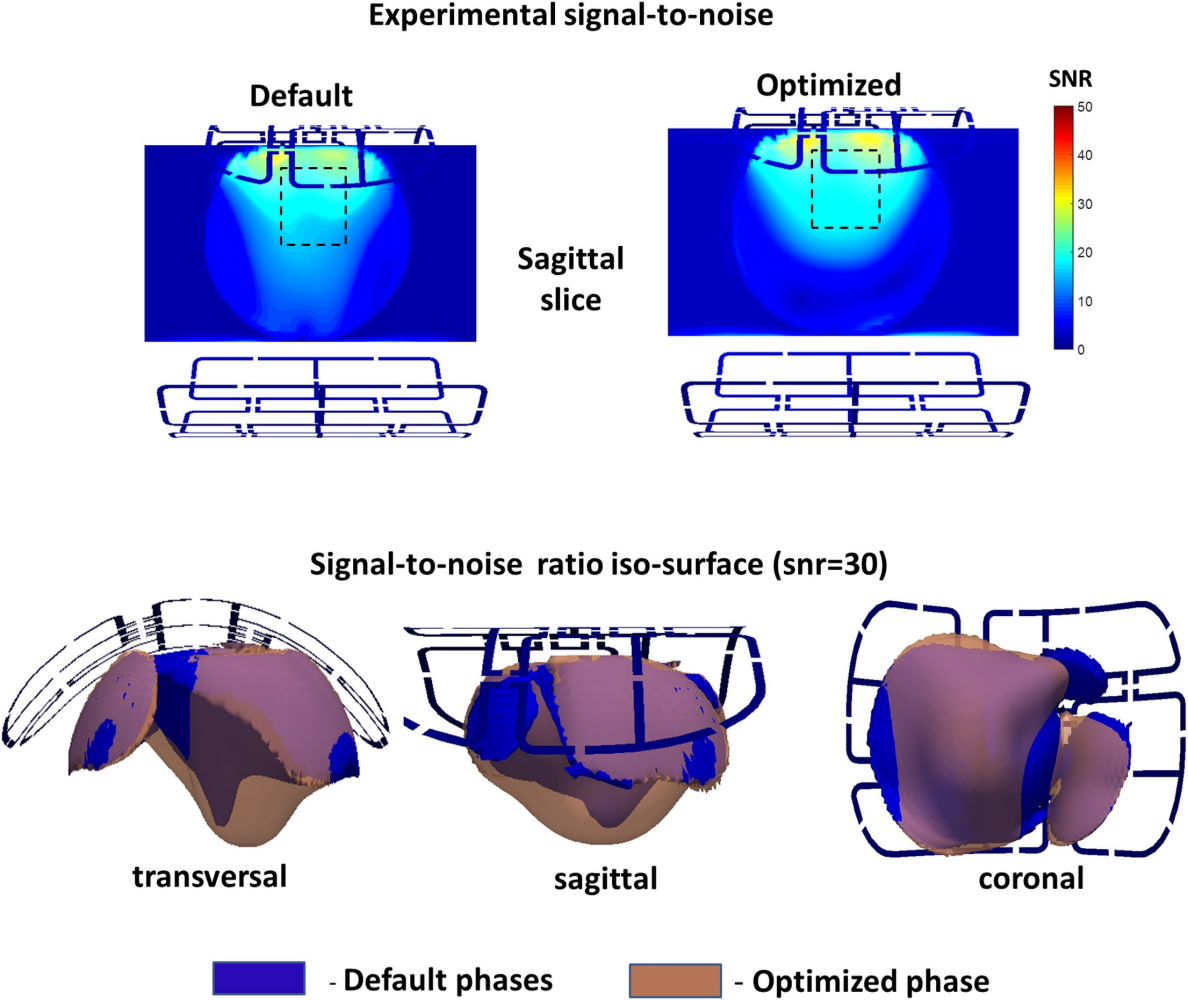
GRE MR-images acquired using phase vector settings optimized for N = 8 channels in P_1_ phantom for default and optimized phase vectors. SNR map with labeled optimization region is shown on panel (a). Panel (b) demonstrates iso-surface labeling volume of SNR>30 for images acquired before and after application of the optimized vector.

Fig 10A and 10B show the GRE MR-images acquired using phase vector settings optimized for N = 8 channels in P_1_ phantom. Phases vector were set using the pTX adjustment platform of the scanner simulating usage of the array in the “pTX Compatibility Mode”. The mean SNR value in the optimized coronal region is improved by ≈60%. For the labeled on the panel (a) optimization region the ≈40% increase of the volume with SNR>30 was achieved as shown on panel (b). The average local gradient of SNR in the anterior-posterior direction was reduced by ≈50%.

**Fig 11:**
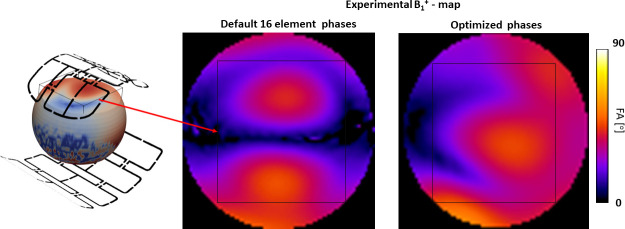
Result of optimization of 16 components phase vector for the phantom P_2_. **B**_**1**_^**+**^**-map acquired in the central coronal slice of the optimization ROI labeled on the left panel.** The center and right panels show B_1_^+^-maps before and after setting optimized phases respectively.

Fig 11 shows the experimental result of the optimization of 16 components phase vector for the phantom P_2_. An optimized phase vector for driving voltages of array elements was set using phase shifters (coaxial cables) connected to the dedicated sockets of the RF-interface. B_1_^+^-map acquired in the central coronal slice of the optimization ROI labeled on the left panel. The central panel and right panels show B_1_^+^-map before and after setting optimized phases.

## Discussion

In this paper, we demonstrated a relatively simple way to balance the computation time with the depth of optimization search targeted to reach the best possible homogeneity of the combined B_1_^+^-field achievable by the fine-tuning of the array by the developer before (or without) subject-specific adjustments in the scanner. The efficiency of the approaching to a globally optimal for the formulated optimization problem phase vector is expected to be close to the full phase space exhaustive search, however, achieved with a realistic computation time even for high-dimensional problems (N>16). To the best of our knowledge, the groups reporting the development of in-house designed transceiver arrays for UHF MRI usually use implicit or explicit pragmatic limitations on the depth of exploring of possible phase combinations in their approaches to the adjustment of the default phases. The results of this work demonstrate that the arbitrary set limitations on the number of starting vectors for the plain random multi-start optimization may lead to achieving essentially lower B_1_^+^-field homogeneity than using the proposed optimized multi-start strategy with adequately defined phase space raster for Stage I. In particular, with *N*_*start*_ = 1000 starting vectors and *N* = 8 optimized phase components the random multi-start optimization using the cost function based on the coefficient-of-variation may lead to the relative gradient which is by factor 10 higher compared to the one achieved in the optimized multi-start. The analysis of the high dimensional problem (*N* = 16) confirms that arbitrary set limitations of the starting vectors set may limit the potentially achievable B_1_^+^-homogeneity. For example, as shown by Fig 8, for *N*_*start*_ = 1000, the maximal cost function value achieved by the random-multi-start would be 20% lower than for the described strategy. The increased depth of the random multi-start search (up to the *N*_*start*_ = 7500 initial vectors,) may be still insufficient to reach the same value of the cost function as provided by the proposed strategy with comparable computation time.

The problems of higher dimensions (N = 24..32 elements) should be preferably solved by stepwise iterative optimization. On each step, the dominant array’s elements for specific ROI can be selected, optimized, and combined using found phase vector. This combined section will be considered as a single element for the optimization of the remaining (or next part of) elements as it was proposed in the work [29]. The experimental validation of the optimized phasing by MRI measurements confirms increased uniformity of the B_1_^+^ leading to the improved homogeneity of the SNR in the optimized region. The results of testing the arrays for pig cardiac MRI with default B_1_-profiles optimized using the described technique were demonstrated both ex-vivo and in-vivo for the dedicated pigs arrays [22, 26] and as well as for the human arrays [30, 31]. The limitation of the current approach is that found globally optimal phase settings are valid in the mathematical context of the specific optimization problems i.e. “global maximum/minimum of the specific cost function(s) used in the optimization”. The achieved B_1_ is not necessarily optimal across the “global population” (animals or humans). Moreover, as we demonstrate in the work [30] the human model-based fixed phases optimization is most probably limited to a specific cohort (specific patient weight range and sex). Solution globally optimal to the population of human or animal subjects for the static B_1_-shimming would require using a machine or deep learning approach with a neuronal network trained to a large number of B_1_-maps representing the population [32]. Another approach providing B_1_^+^ optimization which is global regarding a population is a dynamic pTX-based B_1_^+^-shimming with using universal 3D kT pTX-pulses designed based on the multiple B_1_-maps acquired from the population [33]. Both these approaches, however, require the preliminary acquisition of patient-specific B1-maps using a fully operational array validated for SAR safety, whereas the described method is primarily targeted to the optimization at the hardware development and construction stage.

## Conclusion

In this paper, we propose a time-efficient technique for the calculation of globally optimized hardware phases to be used for the construction and the subsequent optimization of transceiver arrays for UHF MRI. The proposed methodology combines the advantages of full coverage of phase space by an exhaustive search with high computational time efficiency of the solver-based optimization. Besides a final hardware adjustment of phases, the proposed approach allows for a rapid evaluating of the B_1_^+^-shimming capability of different array architectures “in silico”. The proposed technique has demonstrated its efficiency in the development of the transceiver arrays with the complex antisymmetric geometry of the elements which does not allow for simple geometry-based guessing of the default hardware phases.

## Supporting information

S1 Fig (a) Cost function computed for the B_1_-fields generated by each of 8 individual Tx-channels combined from the array element pairs as shown by Fig 2B. Panel (b): Cost function *F*_*c*_ computed for the combined B_1c_^+^ of 6 dominant Tx-channels (Tx1-Tx4, Tx6, Tx7) using a fragment of fully sampled phase space grid *G*_*L*_^*N*^ with δϕ = 1°. The numerous local extremums of *F*_*c*_ makes it difficult to find the global optimum using local optimization solver search. Newertheless, the periodicity of *F*_*c*_ provides possibility to find a sufficient number of phase vectors providing the *F*_*c*_ values close to the global maximum (above the dashed line). This can be performed by the a relatively quick brute-force scanning over the phase grid *U*_*L*_^*N*^ randomly sub-sampled from *G*_*L*_^*N*^ with adjustable density factor *s*.
(TIF)

S2 Fig Computation performance of both stages of the optimized multi-start process. In both stages using of GPU brings acceleration of the computations starting from the number of voxels in the optimization region exceeding ~1300. One can notice, that the speed of CPU computation remains practically constant by a 6-fold increase of the optimization volume whereas for CPU computation the time increases linearly up to the number of voxels ~2200 and even faster by larger arrays size. The CPU type used was AMD Ryzen 9 3950x/16 cores. GPU type: GeForce RTX 2080 Titan, 68 multiprocessors, 1.545GHz, Matlab compute capability index = 7.5.
(TIF)
