# Supplementary figures and images for: Global optimization of default phases for parallel transmit coils for ultra-high-field cardiac MRI
Source: PLoS One. 2021 Aug 6;16(8):e0255341. doi: 10.1371/journal.pone.0255341 (PMC8346258; doi:10.1371/journal.pone.0255341)

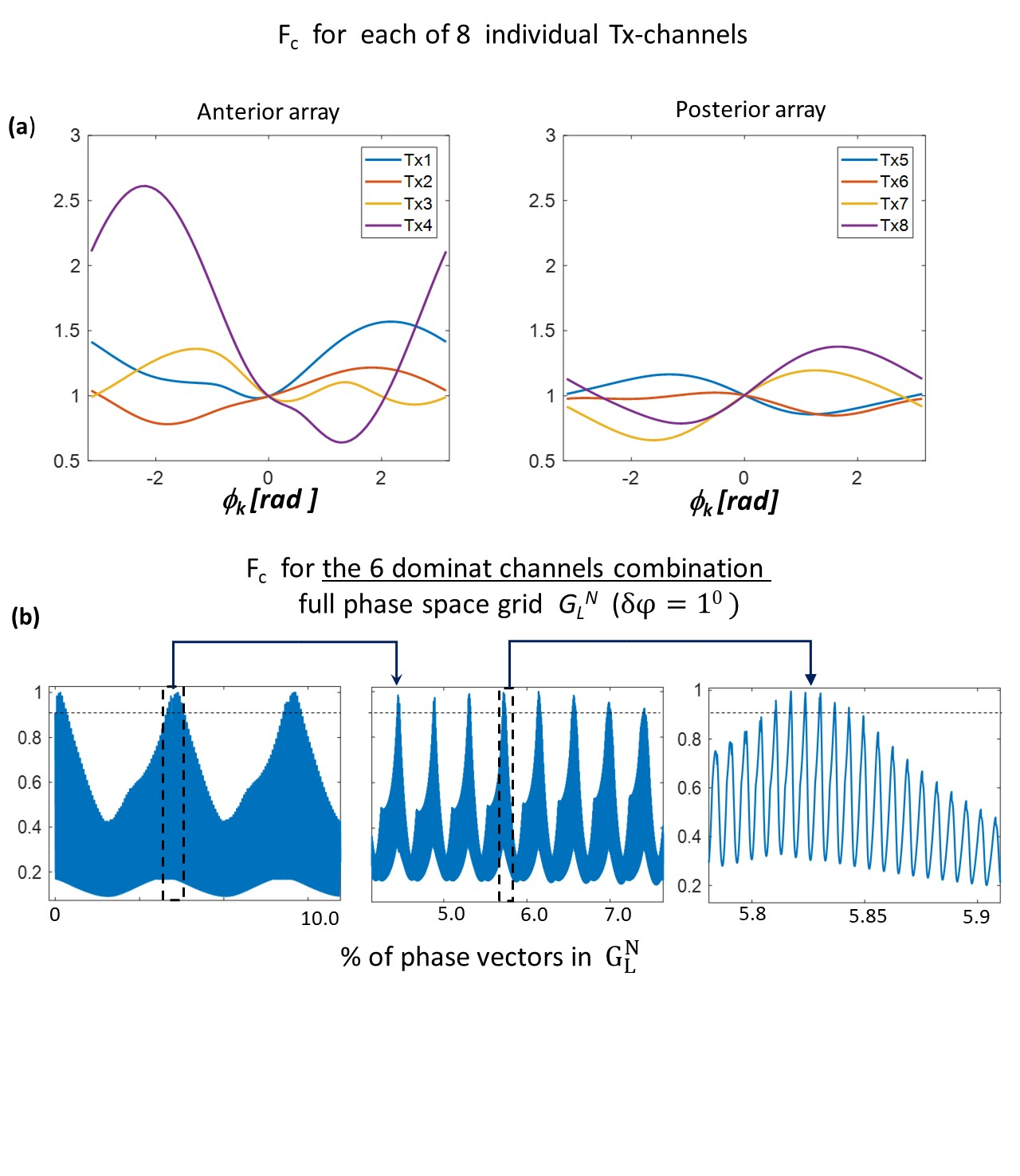

Supplement: S1 Fig — (a) Cost function computed for the B1-fields generated by each of 8 individual Tx-channels combined from the array element pairs as shown by Fig 2B. Panel (b): Cost function Fc computed for the combined B1c+ of 6 dominant Tx-channels (Tx1-Tx4, Tx6, Tx7) using a fragment of fully sampled phase space grid GLN with δϕ = 1°. The numerous local extremums of Fc makes it difficult to find the global optimum using local optimization solver search. Newertheless, the periodicity of Fc provides possibility to find a sufficient number of phase vectors providing the Fc values close to the global maximum (above the dashed line). This can be performed by the a relatively quick brute-force scanning over the phase grid ULN randomly sub-sampled from GLN with adjustable density factor s. (TIF) [file pone.0255341.s001.tif]

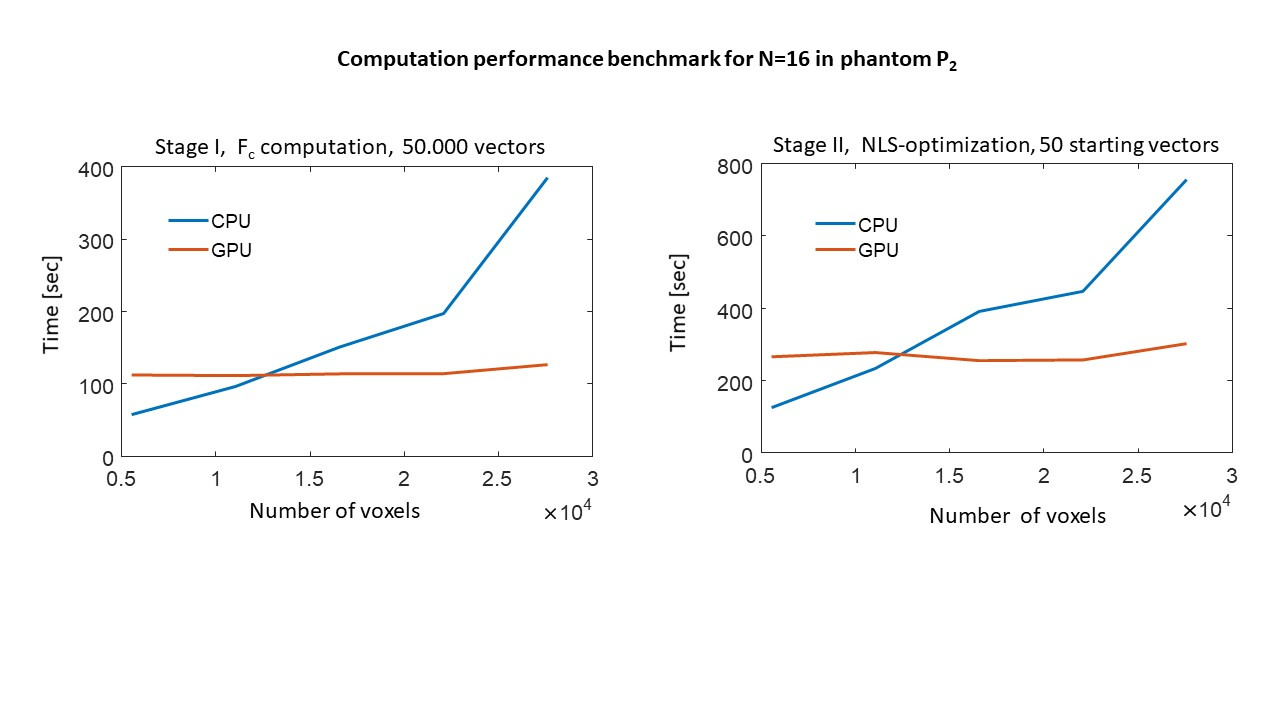

Supplement: S2 Fig — In both stages using of GPU brings acceleration of the computations starting from the number of voxels in the optimization region exceeding ~1300. One can notice, that the speed of CPU computation remains practically constant by a 6-fold increase of the optimization volume whereas for CPU computation the time increases linearly up to the number of voxels ~2200 and even faster by larger arrays size. The CPU type used was AMD Ryzen 9 3950x/16 cores. GPU type: GeForce RTX 2080 Titan, 68 multiprocessors, 1.545GHz, Matlab compute capability index = 7.5. (TIF) [file pone.0255341.s002.tif]
